# The Curability of Insanity and the Individualized Treatment of the Insane

**Published:** 1887-10

**Authors:** 


**The Curability of Insanity and the Individualized Treatment of the Insane.** By JOHN S. BUTLER, M. D., Hartford, Conn., late Physician and Superintendent of the Connecticut Retreat for the Insane, etc. New York and London: G. P. Putnam's Son. 1887.

This little work brings out the author's experience in the treatment of insanity while connected with the Connecticut

Retreat. It aims to emphasize the individualized treatment of mental diseases, based on the physical peculiarities or idiosyncrasies of each case. The work is of special interest to the alienist, to whom we commend it.

